# Castration Instead of Lynching

**Published:** 1906-10

**Authors:** 


### EDITORIAL NOTES AND COMMENTS.

The Business office of THE JOURNAL-RECORD is 1007-1008 Century Building.

The Editorial office is 318-319-320 The Prudential.

Address all Business communications to Dr. M. B. Hutchins, Manager.

Make remittances payable to THE ATLANTA JOURNAL-RECORD OF MEDICINE.

On matters pertaining to the Editorial and Original Communications address Dr. Bernard Wolff, Atlanta.

Reprints of original articles will be furnished at cost price. Requests for the same should always be made *on the manuscript*.

We will present, post-paid, on request, to each contributor of an original article, twenty (20) marked copies of THE JOURNAL-RECORD containing such article.

### CASTRATION INSTEAD OF LYNCHING.

THE epidemic of criminal assault and lynching which for the last few months has been so evident throughout the South, and especially in the vicinity of Atlanta, forces one to the conclusion that the proper remedy is not being utilized in punishing this revolting offense. Instead of having a deterrent effect, it only seems to make matters worse. Many of the offenders go to their death in such a manner as to give the impression to certain of the negroes that they are not criminals but martyrs to race prejudice. Other vicious assaults frequently follow within a few days or a week after a lynching. No matter by what psychological process these are incited, one thing seems certain that they aggravate the danger rather than increase the safety of our wives, mothers and sisters.

In some instances the crime is committed with such brutality that nothing short of immediate death seems sufficient punishment. If, however, by a lynching we increase the danger of other women, should we not hesitate and devise, if possible, a remedy which, instead of making a temporary hero of the negro, will make of him a living example of the punishment to be meted out to all who thus attempt to gratify their bestial lust?

So intense is the animal passion of the negro that to be deprived of his sexual power would of necessity make him an object of ridicule and contempt among them.

In animal life we see clearly in the tractable gelding, the castrated mule, the ox, the capon, and in fact, in all animals and fowls, the effect of castration. In man, except in very rare instances, sexual power is lost by the removal of the testicles, and along with it he becomes docile, quiet and inoffensive. After castration his disgrace would be lasting in its moral effect.

To persuade an incensed mob that such punishment is preferable to lynching would be difficult until they could be shown the effect of emasculation, and that the negro would rather be hanged than to be deprived of sexual power so dear to him.

The kuklux klan, recently agitated, could be very useful in taking charge of these affairs in a regular way, and in assuring the frenzied friends that a punishment worse than death will be inflicted. The identity of the criminal could be ascertained by an orderly trial by the klan, and in this manner avoid punishing an innocent negro, as is often done in lynchings. The trial could be made more or less mysterious and impressive, for the negro stands in great awe of mysteries. This was clearly shown in Atlanta last winter by the belief among the negroes that there was a black wagon driven through the streets at night to capture subjects for the dissecting-rooms of the medical colleges. Absurd as is the idea, it made a profound impression upon the negroes and even those more or less intelligent were afraid to venture out on the darker streets at night.

An impressive trial by a ghost-like kuklux klan and a "ghost" physician or surgeon to perform the operation would make of it an event the "patient" would never forget, nor cease to talk about and enlarge upon. This would do away with the martyrdom effect of lynching as well as the demoralizing results of mob law. The badge of disgrace and emasculation might be branded upon the face or forehead, as a warning, in the form of an "R," emblematic of the crime for which this punishment was and will be inflicted.

As long as many of the negroes believe, regardless of the revolting character of the crime, that lynchings are the result of race prejudice, this mode of punishment will only incense them and aggravate the condition. Certain of them think their cause will be strengthened by these (according to them) martyrs just as the Christian church spread more rapidly after the persecution by Nero.

All will admit that lynching is not only a failure, but that it is dangerous. Castration in no particular could be worse.

---

MEMBERS of the profession are warned against the operations of one C. E. Simpson, who is fraudulently taking orders for *Surgery Gynecology and Obstetrics,* published by the Surgical Publishing Company of Chicago, and under the managing editorship of Franklin H. Martin, M.D.

Many doctors have already been victimized by this man to the extent of paying cash for orders for the journal, or giving him checks payable to his own order; and this notice is published in the interest of the profession and for the purpose of putting a stop to his further operations. Secretaries of local medical societies are requested to warn the members of their societies against him.

